# Deficiency of myotubularin-related protein 14 influences body weight, metabolism, and inflammation in an age-dependent manner

**DOI:** 10.1186/s13578-015-0062-6

**Published:** 2015-12-21

**Authors:** Yin Lv, Lu Xue, Congli Cai, Qing-Hua Liu, Jinhua Shen

**Affiliations:** Institute for Medical Biology and Hubei Provincial Key Laboratory for Protection and Application of Special Plants in Wuling Area of China, College of Life Sciences, South-Central University for Nationalities, 182 MinZu Ave, Wuhan, 430074 Hubei China; Wuhan Youzhiyou Biopharmaceutical Co., Ltd., Wuhan, 430075 China

**Keywords:** Phosphatase MTMR14, Metabolism, Inflammation, PI3K/AKT and ERK signaling pathways, Adult obesity

## Abstract

**Background:**

Myotubularin-related protein 14 (MTMR14) is a novel phosphoinositide phosphatase with roles in the maintenance of normal muscle performance, autophagy, and aging in mice. Our initial pilot study demonstrated that MTMR14 knock out (KO) mice gain weight earlier than their wild-type (WT) littermates, which suggests that this gene may also be involved in metabolism regulation.

**Results:**

The present study evaluated the role of MTMR14 in the development of aging-associated obesity. We found that aged MTMR14 KO mice fed a normal chow diet exhibited increased serum triglyceride, total cholesterol, and glucose levels compared to age-matched WT controls. Lipid accumulation was also increased in aged KO mice. Several inflammatory cytokines and adipokines were dramatically dysregulated in the metabolic tissues of aged MTMR14 KO mice compared to control mice. Circulating inflammatory cytokines were significantly elevated and plasma adipokine levels were abnormally regulated in aged MTMR14 KO mice. These data suggest that MTMR14 deficiency caused a late-onset inflammation and metabolic dysfunction. Further study demonstrated that this exacerbated metabolic dysfunction and inflammation may be regulated by the phosphoinositide 3 kinase/protein kinase B and extracellular signal-regulated protein kinase signaling pathways.

**Conclusions:**

Our current research suggests that MTMR14 deletion induces overweight and adult obesity accompanied by chronic inflammation in an age-dependent manner.

## Background

In 2014, the World Health Organization (WHO) has predicted that approximately 2–3 billion adults will be overweight; 700 million adults will be obese, and 200 million school-aged children will be obese/overweight [1]. The global increase in the prevalence and incidence of obesity has drawn attention to this issue as a major public health concern. Obesity is commonly attributed to increased body weight, accumulated fat, metabolic complications, and chronic systemic inflammation [2, 3]. Obesity is associated with various chronic diseases, including metabolic syndrome, cardiovascular diseases, diabetic retinopathy, respiratory disease, and cancer [4]. It is widely accepted that the cause of the obesity epidemic is a consequence of rapid changes in environment and lifestyle, but it is not clear why some individuals are more susceptible to an obesogenic environment than others [5, 6]. The major risk factors for obesity are environmental and genetic, and several candidate genes are involved in obesity in mice and humans, including glucose transporter type 4 (Glut4), leptin, adiponectin, tumor necrosis factor (TNF-α), interleukin 6 (IL-6), interleukin 1β (IL-1β), monocyte chemotactic protein 1 (MCP-1), glucose-6-phosphate (G6P), and phosphoenolpyruvate carboxykinase (PEPCK) [1, 7–11].

Myotubularin-related protein 14 is a novel phosphoinositide phosphatase. An inactivation mutation of MTMR14 was first identified in human centronuclear myopathy in 2006 [12, 13], suggesting that this gene is involved in muscle disease. Deletion of MTMR14 in mice disrupts calcium homeostasis and causes a muscle disorder [14]. MTMR14 is also involved in the regulation of autophagy and aging [15–19]. Our recent work revealed that MTMR14 KO mice weighed more than their WT littermates as adults, especially aged mice, which suggests that MTMR14 is involved in the regulation of body weight and metabolism.

We used MTMR14 KO male mice as a working model to investigate the mechanism of MTMR14 in obesity. A series of physiological indexes demonstrated that the loss of MTMR14 induced obesity in an age-dependent manner, as reflected by body weight, energy intake and expenditure, blood biochemical indexes, and fat accumulation. Further research demonstrated that the PI3K/AKT and ERK signaling pathways are involved in MTMR14 deletion-regulated obesity.

## Results and discussion

### MTMR14 KO mice got fat earlier than WT mice

**Fig. 1:**
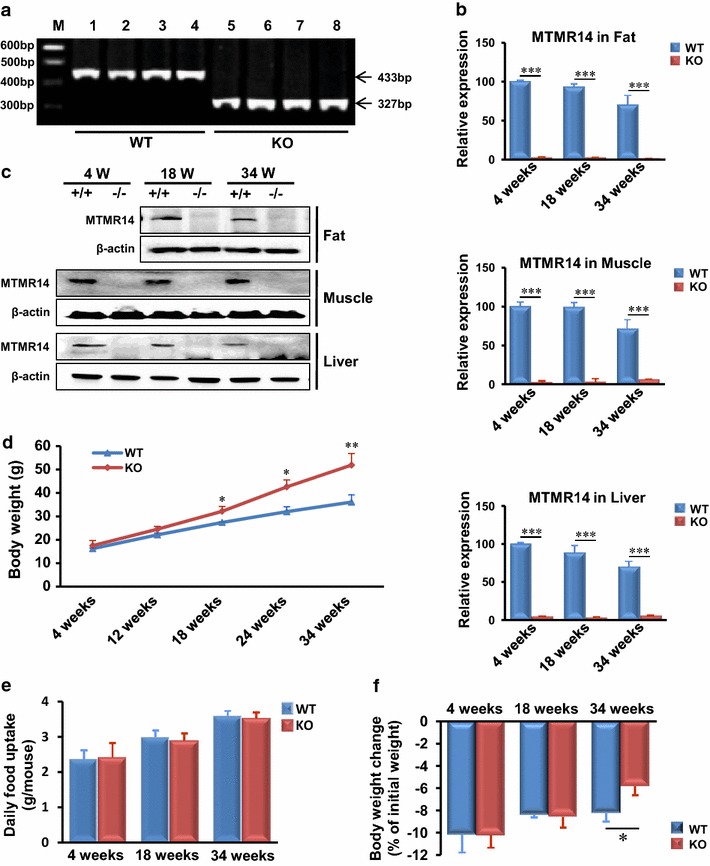
Growth curve, energy intake and energy expenditure of WT and MTMR14 KO mice. Mice in these experiments were fed a standard chow diet. **a** PCR genotyping results of WT and MTMR14 KO mice. **b**, **c** MTMR14 mRNA and protein expression levels in three metabolic tissues of WT and KO mice at different ages (4, 18 and 34 weeks), respectively. **d** The growth curve of WT and MTMR14 KO mice. Body weights of WT and MTMR14 KO mice were measured at 4, 12, 18, 24, and 34 weeks of age. **e** The daily food intake of WT and MTMR14 KO mice was measured in an age-matched manner (4, 18 and 34 weeks). **f** The amount of 12 h fasting-induced body weight change in WT and MTMR14 KO mice was calculated as an approximation of energy expenditure. The values are expressed as the mean ± SD. *p < 0.05, **p < 0.01, n = 8

Our previous results demonstrated that MTMR14 KO mice were born at the expected Mendelian ratio and did not exhibit obvious abnormalities [14]. WT and MTMR14 KO mice were identified through PCR genotyping. As we expected, MTMR14 mRNA and protein were almost undetectable in the metabolic tissues of male KO mice at different ages (Fig. 1b, c). Then the growth curve, daily energy intake and expenditure were measured at different time points of male WT and MTMR14 KO mice fed a normal chow diet (Fig. 1d–f; Table 1). Growth curve analyses revealed that genotype and age significantly affected weight (*p* < 0.001). A significant interaction of genotype and age was also observed (*p* < 0.001). Figure 1d shows that no large differences were recorded when the mice were younger than 12 weeks. However, MTMR14 KO mice had more body fat than the WT controls after 12 weeks (*p* < 0.05), and the difference was more dramatic (*p* < 0.01) with increased age. We measured daily energy intake and expenditure in age-matched WT and MTMR14 KO mice (Fig. 1e, f). Daily food intake revealed no significant difference between WT and MTMR14 KO mice (Fig. 1e). Further analyses of energy expenditure demonstrated that aged MTMR14 KO mice lost less body weight than WT mice (Fig. 1f). These results indicated that aged MTMR14 KO mice developed obesity as a result of reduced energy expenditure.

**Table 1:** Metabolic and inflammatory variables in MTMR14 WT and KO mice

Variables	WT			KO		
	4 weeks	18 weeks	34 weeks	4 weeks	18 weeks	34 weeks
Body weight (g)	16.2 ± 0.2	27.4 ± 0.3	36.1 ± 3.1	17.5 ± 2.3	32.2 ± 2.1*	51.9 ± 4.9**
Daily food intake (g/mouse)	2.37 ± 0.14	2.98 ± 0.19	3.57 ± 0.15	2.41 ± 0.41	2.88 ± 0.22	3.51 ± 0.16
Body weight change (% of initial weight)	−10.16 ± 1.6	−8.4 ± 0.2	−8.26 ± 0.7	−10.23 ± 1.1	−8.54 ± 1.0	−5.84 ± 0.8*
TG (mg/dL)	96.49 ± 18.54	106.35 ± 19.80	115.78 ± 14.22	108.25 ± 17.64*	112.45 ± 16.73*	124.26 ± 23.61*
TC (mg/dL)	88.78 ± 14.43	91.57 ± 16.72	95.59 ± 17.35	87.35 ± 12.62	94.68 ± 14.24	106.93 ± 18.16 *
Blood glucose (0 min) (mg/dL)	69 ± 4	53 ± 4	61 ± 8	77 ± 13	72 ± 6*	81 ± 5*
Blood glucose (15 min) (mg/dL)	229 ± 25	224 ± 22	172 ± 8	243 ± 24	220 ± 11	251 ± 13**
Blood glucose (30 min) (mg/dL)	202 ± 11	206 ± 17	155 ± 11	210 ± 19	221 ± 23	208 ± 22*
Blood glucose (60 min) (mg/dL)	176 ± 14	166 ± 18	116 ± 10	178 ± 20	186 ± 8	157 ± 12*
Blood glucose (120 min) (mg/dL)	141 ± 6	107 ± 4	91 ± 13	139 ± 13	130 ± 14	124 ± 11*
Fat mass/body weight (mg/g)	20.82 ± 0.91	30.42 ± 0.79	22.96 ± 1.29	21.46 ± 0.82	31.23 ± 0.84	27.48 ± 1.22**
TNF-α (pg/mL)	1168 ± 184	1331 ± 146	1705 ± 182	1207 ± 193	1428 ± 173	3407 ± 289***
IL-6 (pg/mL)	176 ± 30	215 ± 13	316 ± 13	180 ± 31	210 ± 42	556 ± 107**
Leptin (pg/mL)	6615 ± 409	7150 ± 522	8678 ± 425	5767 ± 401	9025 ± 735	20436 ± 422***
Adiponectin (ng/mL)	379 ± 48	384 ± 64	308 ± 47	350 ± 29	329 ± 34	141 ± 27***

Body weight, daily food intake, body weight change, TG, TC, blood glucose level, fat mass/body weight, plasma TNF-α, IL-6, leptin and adiponectin of age-matched WT and KO mice were evaluated. Data are mean ± SD of 7–8 mice per group. All WT and KO data were statistically analyzed in an age-matched manner

* p < 0.05, ** p < 0.01, *** p < 0.001 vs. the respective WT group

### MTMR14 KO mice exhibited increased serum TG, TC, and glucose levels in an age-dependent manner

Obesity arises from an imbalance in energy intake and expenditure that eventually leads to the pathological growth of adipocytes [20]. We monitored serum TG, TC, and blood glucose levels and glucose tolerance to clarify the possible metabolic complications associated with the obese phenotype in the aged MTMR14 KO mice. MTMR14 KO mice exhibited a marked elevation in fasting serum TG levels at an earlier age (4 weeks) compared to control mice. Fasting TG levels in MTMR14 KO mice were sustained higher than age-matched WT controls after 30 weeks of feeding a normal chow diet (Table 1). No distinguishable differences in serum TC levels were recorded when the mice were young (≤18 weeks), but fasting serum TC levels were significantly higher in MTMR14 KO mice at 34 weeks of age than controls (Table 1).

**Fig. 2:**
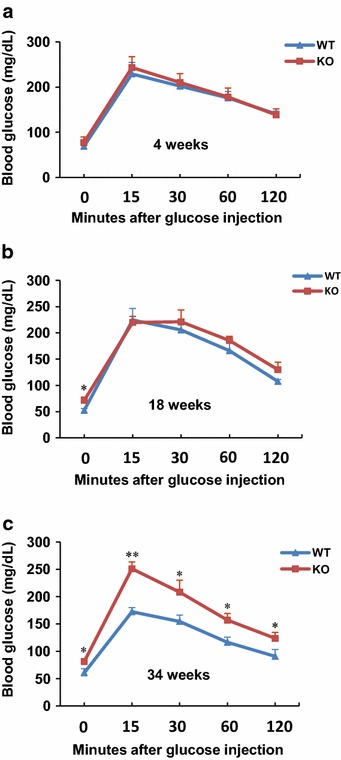
Glucose tolerance in WT and MTMR14 KO mice. **a**–**c** Glucose tolerance tests were performed in WT and MTMR14 KO mice at 4, 18 and 34 weeks. For GTT experiments, 2 g d-glucose/kg body weight was administered to 12-h fasted WT and MTMR14 KO mice in an age-matched manner, and blood glucose levels were sampled from venous tail blood at the indicated time-points. *p < 0.05, **p < 0.01, n = 7

Glucose tolerance tests (GTTs) were performed in 4-week-, 18-week- and 34-week-old WT and MTMR14 KO mice (Fig. 2; Table 1). Statistical analysis revealed that genotype and age significantly affected blood glucose level (p < 0.001). A significant interaction of genotype and age was observed (p < 0.001). In aged mice (34 weeks), genotype and minutes after injection both significantly affected blood glucose level (p < 0.001), A significant interaction of genotype and minutes after injection was also observed (p < 0.01). Glucose level and tolerance in MTMR14 KO mice were not significantly different after an exogenous glucose challenge at an early age (4 weeks) compared to WT littermates (Fig. 2a). Similarly, fasting glucose levels were almost undistinguishable after glucose injections in the adult test group (18 weeks). However, basal glucose levels were significantly higher in 18-week-old MTMR14 KO mice than WT controls (Fig. 2b). The most severe glucose intolerance was observed with aging (Fig. 2c). In aged MTMR14 KO mice (34 weeks), higher glucose levels were observed compared with WT mice in 0, 15, 30, 60, 120 min after injection, respectively. These results suggest that MTMR14 deficiency impairs glucose and lipid metabolism even in mice fed a normal chow diet and that the metabolic disorders become severe with aging.

### MTMR14 KO mice exhibit exacerbated fat accumulation

**Fig. 3:**
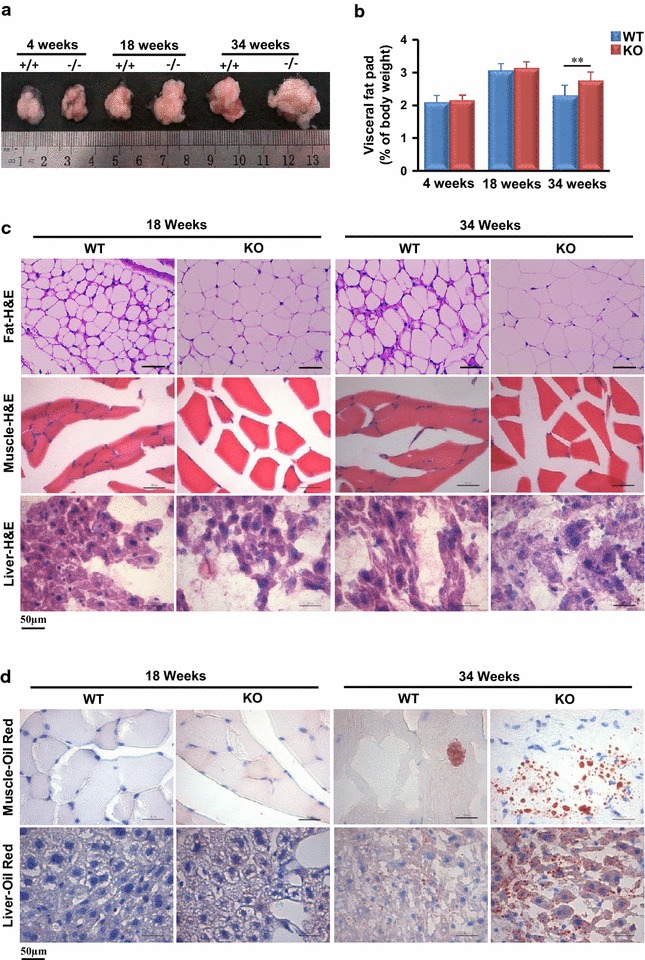
MTMR14 deletion exacerbated fat accumulation. **a** Pictures of visceral fat pad from WT and MTMR14 KO male mice at 4, 18, and 34 weeks of age. **b** The fat mass (% of body weight) in WT (*blue*) and MTMR14 KO (*red*) male mice at different ages. **c** metabolic tissues (fat, muscle, liver) of WT and MTMR14 KO male mice at 18 weeks old or 34 weeks old was collected and stained with hematoxylin-eosin (H&E). **d** Histology of fat, muscles and livers of WT and MTMR14 KO male mice using Oil Red O staining. Pictures were taken under ×400 magnifications

Fat accumulation and adipocyte differentiation are associated with the occurrence and development of obesity [10]. As shown in Fig. 3a, histological analysis revealed that there were no obvious differences in fat accumulation between 4-week-old KO and WT mice. The livers of 18-week-old MTMR14 KO mice exhibited a subtle accumulation of visceral adipose tissue compared to WT littermates, and an obvious lipid accumulation was observed in 34-week-old WT mice. In contrast, MTMR14 KO mice exhibited a dramatically exacerbated accumulation of visceral adipose tissue (Fig. 3a). The visceral fat pad (% of body weight) exhibited no significant differences in young mice (≤18 weeks), but the percentage in aged MTMR14 KO mice was significantly increased compared to that in the WT control (Fig. 3b; Table 1). Further histological analyses of metabolic tissues revealed that the size of adipocytes in aged MTMR14 KO mice increased compared to those in WT mice (Fig. 3c). More lipid droplets accumulated in the myocytes and hepatocytes of 34-week-old MTMR14 KO mice (Fig. 3d), and hepatic steatosis was also observed (Fig. 3c, d). These data demonstrated that MTMR14 deficiency promoted hepatic steatosis, lipid droplet accumulation and increased adipocyte size in aged MTMR14 KO mice.

### MTMR14 deficiency down-regulated metabolism-associated factors and up-regulated inflammation-related gene expression in the fat tissue of aged mice

**Fig. 4:**
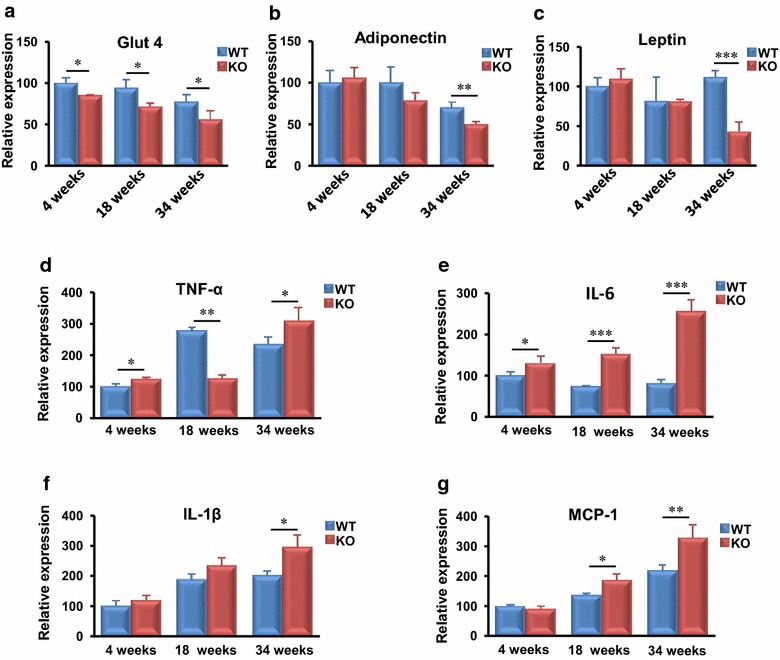
MTMR14 deficiency down-regulated metabolism-associated genes but up-regulated inflammation-associated gene expression with age in fat. **a**–**c** The expression levels of mRNAs related to metabolism (Glut4, adiponectin, leptin) and **d**–**g** inflammation (TNF-α, IL-6, IL-1β, MCP-1) in the fat of WT (*blue*) and MTMR14 KO (*red*) mice at different ages (4, 18, and 34 weeks) were detected. *p < 0.05, **p < 0.01, ***p < 0.001

Fat and muscle are important tissues for energy storage and utilization. Glut4, adiponectin, and leptin are important genes that play positive roles in the regulation of obesity and body metabolism [12, 21, 22]. We investigated whether the expression of these genes was affected in MTMR14 KO mice. The expression level of Glut4 decreased in MTMR14 KO mice at every stage compared to that in their WT littermates (Fig. 4a). Adiponectin and leptin mRNA expression was only reduced in aged MTMR14 KO mice (Fig. 4b, c). Obesity is a systemic, low-grade, chronic inflammatory reaction. Therefore, we examined the expression of typical pro-inflammatory cytokines, including TNF-α, IL-6, IL-1β, and MCP-1 [23]. The mRNA level of TNF-α was elevated in young and aged MTMR14 KO mice compared to WT controls (Fig. 4d), and IL-6 exhibited a higher expression level in MTMR14 KO mouse fat at every age (Fig. 4e). The mRNA expression of IL-1β was also increased in older MTMR14 KO mice (Fig. 4f), and the expression level of MCP-1 was significantly increased in adult MTMR14 KO mice (Fig. 4g).

### MTMR14 deficiency down-regulated metabolism-associated factors and up-regulated inflammation-related gene expression in the muscle of aged mice

**Fig. 5:**
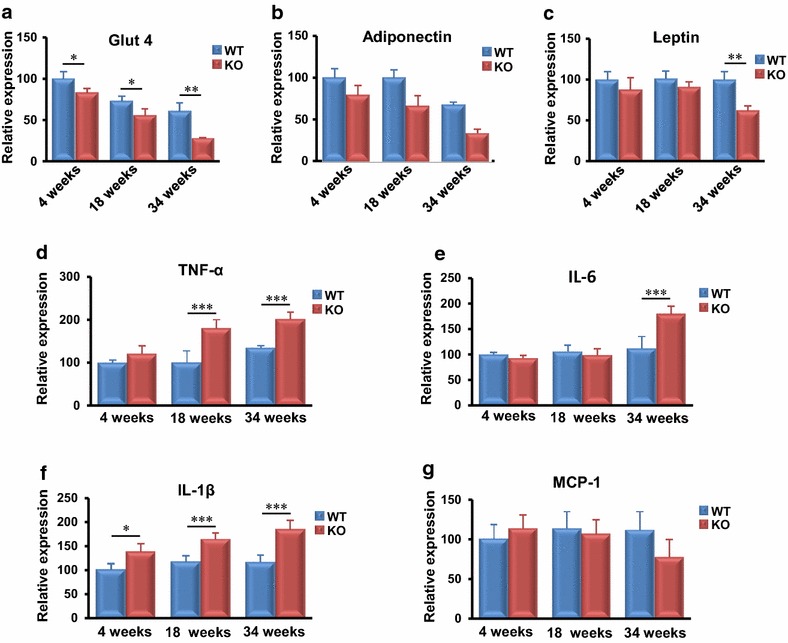
MTMR14 deficiency down-regulated metabolism-associated genes but up-regulated inflammation-associated gene expression with age in muscle. **a**–**g** Real-time PCR analysis of Glut4, adiponectin, leptin, TNF-α, IL-6, IL-1β, and MCP-1 mRNA levels in muscles. *p < 0.05, **p < 0.01, ***p < 0.001

Quantitative PCR of muscle demonstrated that the expression levels of Glut4 and leptin were lower in MTMR14 KO mice of every age (Fig. 5a, c). Adiponectin expression in adult MTMR14 KO mice also decreased (Fig. 5b). In contrast to adipokines, inflammatory cytokine expression was up-regulated in the muscle of MTMR14 KO mice (Fig. 5d–f). MCP-1 was not significantly different in MTMR14 KO mice at any age (Fig. 5g).

### MTMR14 deficiency dysregulated metabolism and inflammation-associated gene expression in aged mouse liver

**Fig. 6:**
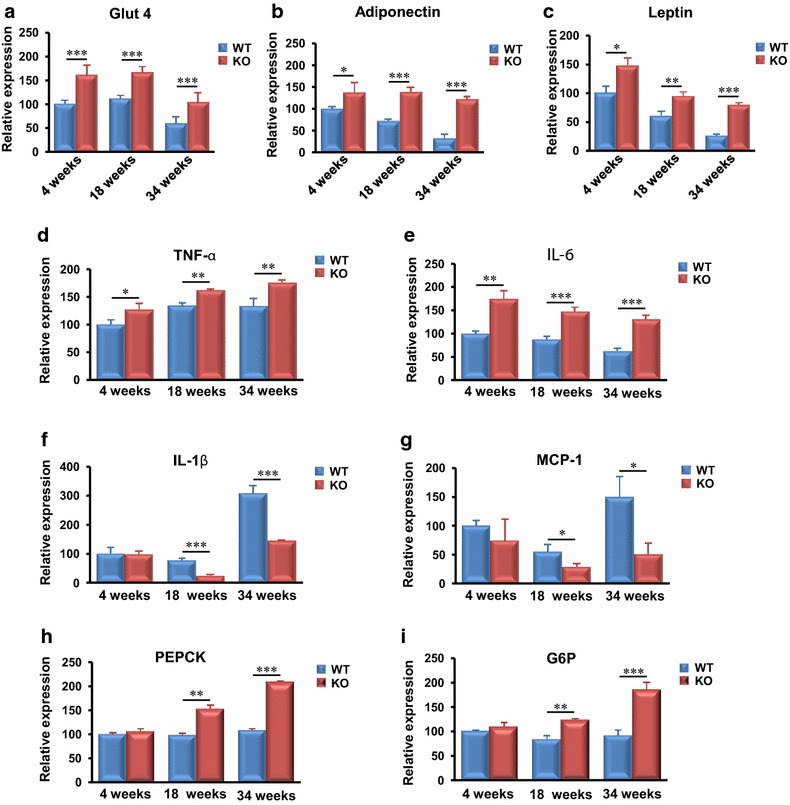
MTMR14 deficiency up-regulated metabolism- and inflammation-associated gene expression with age in the liver. **a**–**i** The expression levels of Glut4, adiponectin, leptin, TNF-α, IL-6, IL-1β, MCP-1, PEPCK and G6P in the livers of WT blue) and MTMR14 KO (red) mice at different ages (4, 18, and 34 weeks) were detected. *p < 0.05, **p < 0.01, ***p < 0.001

The liver plays a vital role in metabolism and detoxification. Figure 6 shows that adipokine mRNA levels increased dramatically in the livers of MTMR14 KO mice (Fig. 6a–c). We also found that TNF-α and IL-6 mRNA levels were increased in MTMR14 KO mice even at an early age (Fig. 6d, e). IL-1β and MCP-1 mRNA levels were indistinguishable between WT and MTMR14 KO mice at an early age (4 weeks), but these levels decreased in aged MTMR14 KO mice (≥18 weeks) (Fig. 6f, g). PEPCK and G6P, which produce insulin-responsive enzymes of hepatic glyceroneogenesis [24], exhibited significantly higher mRNA levels in MTMR14 KO mice at 18 weeks, and these levels were sustained at 34 weeks (Fig. 6h, i).

These results demonstrate that the loss of MTMR14 promoted metabolic dysfunction and inflammation in fat, muscle and liver, particularly in aged mice.

### MTMR14 deficiency altered serum inflammatory cytokine and adipokine expression

**Fig. 7:**
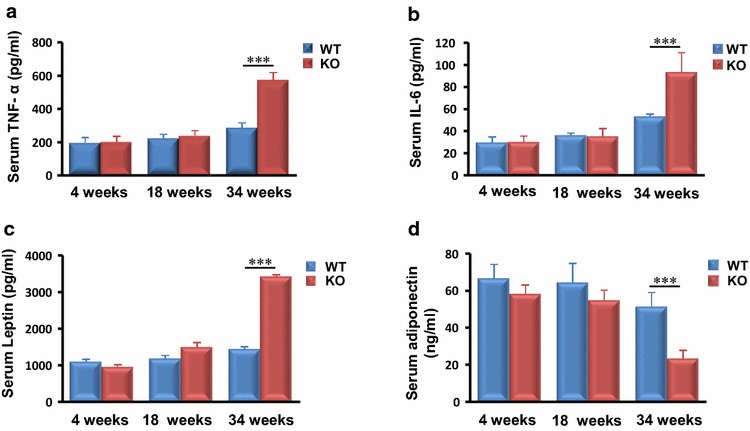
MTMR14 deficiency influenced plasma concentrations of inflammatory cytokines and adipokines. **a**–**d** The plasma levels of TNF-α, IL-6, leptin and adiponectin in WT (*blue*) and MTMR14 KO (*red*) mice were measured in an age-matched manner (4, 18, and 34 weeks). **p < 0.01, ***p < 0.001

Figures 4, 5 and 6 show that MTMR14 deletion evoked a dysregulation of several metabolic- and inflammation-associated genes in fat, muscle and liver. Plasma inflammatory cytokine and adipokine levels were measured to examine the overall influence of abnormal expression patterns in metabolic tissues (Fig. 7; Table 1). Circulating inflammatory cytokines, TNF-α and IL-6 were dramatically increased only in aged MTMR14 KO mice (Fig. 7a, b). Plasma leptin levels were also elevated in aged MTMR14 KO mice (Fig. 7c), but adiponectin exhibited an obvious reduction in these mice compared to their WT littermates (Fig. 7d). Plasma leptin and adiponectin levels were not significantly different in young mice (≤18 weeks). These results indicated that MTMR14 deficiency may lead to late-onset inflammation and abnormal metabolism.

### MTMR14 deficiency dysregulated PI3K/AKT and ERK signaling pathways in adipose tissue, muscle, and liver

**Fig. 8:**
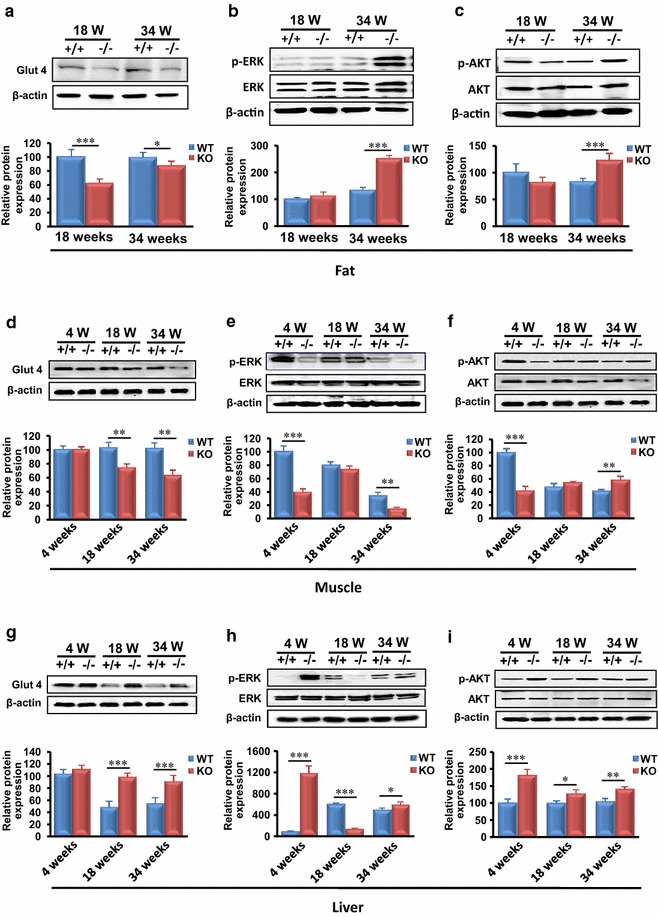
MTMR14 deficiency altered PI3K/AKT and ERK signaling pathways in fat, muscle and liver. **a** Expressions levels of Glut4 in fat. **b, c** The phosphorylation levels of ERK and AKT in fat of WT and MTMR14 KO mice at different ages (4, 18, and 34 weeks) were detected. **d** The expression levels of Glut4 in muscles of MTMR14 WT and KO mice. **e, f** The phosphorylation levels of ERK and AKT in muscles of WT and MTMR14 KO mice at different ages (4, 18, and 34 weeks) were detected. **g** The expression levels of Glut4 in the livers of MTMR14 WT and KO mice. **h, i** The phosphorylation levels of ERK and AKT in livers of WT and MTMR14 KO mice at different ages (4, 18, and 34 weeks) were detected. Quantitative measurements of Glut4, p-ERK, p-ARK proteins in fat, muscle and liver tissue of WT (*blue*) and KO (*red*) mice are shown underneath the original pictures. *p < 0.05, **p < 0.01, ***p < 0.001. β-actin was included as a control

The phosphoinositide 3 kinase/protein kinase B (PI3K/AKT) and extracellular signal-regulated protein kinase (ERK) signaling pathways are important in obesity, inflammation and associated chronic diseases. The activation of two critical protein kinases in PI3K/AKT and ERK signaling pathways, ERK and AKT, plays an important role in cell cycle, proliferation, apoptosis, and autophagy [25–28]. The current study investigated ERK and AKT phosphorylation in three tissues of MTMR14 KO and WT mice. In fat, Glut4, which is a vital glucose transporter in insulin response, was decreased in adult and aged MTMR14 KO mice (Fig. 8a) and the phosphorylation level of ERK (p-ERK) was increased in aged MTMR14 KO mice (Fig. 8b). An elevated phosphorylation of AKT (p-AKT) in fat tissue in aged MTMR14 KO mice was also observed (Fig. 8c).

In muscle, the expression of Glut4 was significantly decreased in adult and aged MTMR14 KO mice compared with WT littermates (Fig. 8d). Similarly, p-ERK protein levels were significantly reduced in muscle tissue in 4-week-old and 34-week-old MTMR14 KO mice (Fig. 8e), and the levels of p-AKT were increased in KO mice at 18 and 34 weeks (Fig. 8f).

In liver tissue, the expression of Glut4 was markedly increased in adult and aged MTMR14 KO mice (Fig. 8g). p-ERK levels were increased in liver tissue in young and aged MTMR14 KO mice, but p-ERK was reduced at 18 weeks (Fig. 8h). AKT phosphorylation was sustained at a higher expression level in liver tissue in MTMR14 KO mice at every age compared to WT littermates (Fig. 8i).

These data demonstrated that MTMR14 deficiency evoked dysfunction in the PI3K/AKT signal pathway. However, the detailed mechanisms require further investigation.

## Discussion

Energy imbalance exerts a negative effect on body conditions and evokes numerous health problems, including diabetes, cardiovascular diseases and cancer. Current research focuses on certain people who seem healthy with normal body weight and limited physiological symptoms at a young age but develop extra body fat and severe blood chemical indexes as adults, which leads to inflammation and metabolic dysfunction (i.e., “adult obesity”). Adult obesity has a negative influence on people’s health, work and quality of life, and it creates a huge financial burden on government medical care systems. Recent research has indicated that excess calorie intake, little physical exercise and obesity genes are important factors contributing to obesity.

MTMR14 is a newly identified phosphoinositide phosphatase that was first identified in human autosomal centronuclear myopathy [12]. MTMR14 favors a variety of phosphatidylinositol phosphates (PIPs) as substrates, such as PI (3, 4) P_2_, PI (3, 5) P_2_, and PI (4, 5) P_2_ [14]. MTMR14 is highly expressed in the heart, muscles and testis, but it is also detected in the kidney, placenta, fat and liver. The functions of MTMR14 primarily include cell autophagy and proliferation, muscle disease and aging [14–16, 18, 19, 29–31]. Our pilot studies found that MTMR14 KO mice accumulate more fat than WT mice in adulthood. However, the exact role of MTMR14 in fat accumulation is largely unknown.

The current study included a series of experiments to delineate the potential role of MTMR14 in obesity. Mice were divided into five major groups based on age: childhood (4 weeks), adolescence (12 weeks), adulthood (18 weeks), middle-aged (24 weeks) and old-aged (34 weeks). A series of physiological indexes were measured in age-matched MTMR14 KO mice and their WT littermates. The results demonstrated that aged MTMR14 KO mice (34 weeks) exhibited significantly higher body weights than WT littermates due to reduced energy expenditure. An increased ratio of fat mass to body weight was also observed in aged MTMR14 KO mice, which was accompanied by higher TC and TG levels, elevated glucose levels, larger adipocytes, and more lipid droplet accumulation in liver and muscle, as revealed by anatomical and histological analyses. These data demonstrated that MTMR14 deletion induced late-onset obesity in mice fed a normal chow diet and exhibited few signs during youth that extra fat would accumulate in adulthood. Obesity also became more severe with age.

Fat, muscle and liver are important tissues that regulate energy balance and metabolism. However, these tissues play diverse roles in physiological processes. Notably, we found that the mRNA expression of inflammatory cytokines and adipokines was specific to metabolic tissue. For example, leptin expression was down-regulated in fat and muscle but up-regulated in liver. The altered expression patterns of adipokines and inflammatory cytokines between liver and fat/muscle may be explained by two hypotheses. First, the different functions of these three tissues are determined by different mRNA expression patterns, but the total organism presented a low-grade, chronic inflammatory state as a result of a comprehensive effect. Serum inflammatory cytokines and adipokines were measured to confirm this hypothesis, and the results demonstrated that circulating TNF-α and IL-6 levels were significantly elevated in aged MTMR14 KO mice compared to age-matched WT controls. These results indicate an increased inflammatory state in aged MTMR14 KO mice. The reduction of adiponectin in aged MTMR14 KO mice demonstrated a severe inflammation and metabolic disorder during aging. Notably, aged MTMR14 KO mice exhibited higher plasma leptin levels, which is consistent with the excessive fat accumulation in these mice. Circulating leptin enables the long-term regulation of fat accumulation via pathways that arise from the hypothalamus [32]. However, our previous finding [14] revealed that MTMR14 was scarce in brain tissue. Therefore, we presumed that MTMR14 plays an indirect role in hypothalamus-mediated metabolic dysfunctions. Taken together, our findings suggest that MTMR14 deficiency evokes severe inflammation via the up-regulation of TNF-α and IL-6, and the metabolic disorders may depend on elevated leptin and decreased adiponectin. Furthermore, the inflammation and abnormal metabolism occurred in an age-dependent manner.

Second, the liver is a vital organ for protein synthesis and metabolic detoxification, and it plays a compensative role [33, 34] when metabolic dysfunction and inflammation impair the normal functions of muscle and fat. However, the decompensation of liver occurred when hepatic steatosis was increased in adult MTMR14 KO mice. The elevated MCP-1 expression level in adipose tissue and increased TNF-α, PEPCK and G6P levels in liver [35] verified the high glyceroneogenesis and hepatic steatosis state in aged MTMR14 KO mice in our study.

**Fig. 9:**
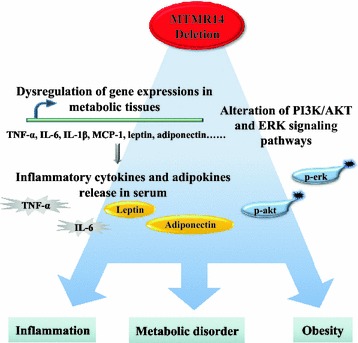
Schema depicting the proposed mechanisms for MTMR14 deletion-induced obesity and complications. On one hand, MTMR14 deletion dysregulates a number of genes then releases a series of inflammatory cytokines and adipokines. On the other hand, MTMR14 deletion alters PI3K/AKT and ERK signaling pathway. As a result, MTMR14 deletion induces obesity and complications

The PI3K/AKT and ERK signaling pathways are vital in numerous inflammation-evoked chronic diseases [36]. Activated PI3K in the PI3K/AKT and ERK signaling pathways produces PI (3,4,5) P_3_ to phosphorylate AKT and regulate ERK phosphorylation [37–39]. Dysfunction of the PI3K/AKT and ERK signaling pathways was observed in the KO mice in the current study. Levels of p-ERK and p-AKT in immature MTMR14 KO mice were down-regulated in muscle but up-regulated in liver compared to WT littermates. The levels of p-ERK were reduced in the liver in adult KO mice, and p-AKT levels were elevated in muscle and liver. The compensative function of the liver was also observed when MTMR14 KO mice were young, but the inflammatory state was not obvious. The expression of p-ERK decreased in muscle and increased in liver and fat with age, but p-AKT was elevated in all three tissues. Generally, MTMR14 deficiency promoted the phosphorylation of AKT and ERK in aged KO mice, and evoked a crosstalk between adipose tissue and inflammatory system, which created inflammation and metabolic disorders. Taken together, as shown in Fig. 9, MTMR14 deletion evokes inflammation, metabolic disorder and obesity by dysregulations of certain vital genes, alterations of PI3K/AKT and ERK signaling pathway and releases of serum cytokines.

Similar to the phosphatase and tensin homolog (PTEN), MTMR14 dephosphorylated several PIPs, which suggests that MTMR14 plays an antagonistic role in the PI3K/AKT and ERK signaling cascades. However, a spatio-temporal dysregulation of p-AKT and p-ERK was observed in our research. A recent report [40] demonstrated that same stimulus (cytokines, inhibitor, serum, siRNA) increased cell-to-cell variations in p-AKT and p-ERK signals, which determined different cell fates. This observation may explain how MTMR14 deletion evoked different tissue-to-tissue expression in different stage and help researchers to draft a p-AKT and p-ERK response map for obesity.

Our previous study demonstrated that MTMR14 deletion impaired calcium homeostasis and elevated calcium concentrations [14]. Recent studies has reported that elevated intracellular calcium could release extracellular inflammatory cytokines and induce obesity and complications [41, 42]. Taken together, MTMR14 deficiency could induce PIP_3_ production-mediated AKT/ERK phosphorylation and ryanodine receptor (RyR) activation-mediated intracellular calcium release. Alterations in the PI3K/AKT and ERK signal pathways, disruptions of calcium homeostasis, and releases of circulating cytokines indicated a state of inflammation and metabolic disorder, which induced obesity and complications. Our previous research also indicated that MTMR14 deletion promote cell autophagy through elevating the phosphorylation levels of AKT and ERK [31]. Recent studies have reported that cell autophagy is closely associated with inflammation, and several inflammatory cytokines, such as TNF-α, IL-6, have been identified as autophagy modulators [43]. Further investigations are needed to figure out the molecular and cellular connections between autophagy and inflammation especially via PI3K/AKT and ERK signaling pathway in MTMR14 deficiency induced chronic diseases.

Notably, this research was performed using normal chow diet. MTMR14 is highly expressed in the heart, and a high-fat diet (HFD) might induce a series of severe cardiovascular diseases in MTMR14 KO mice.

In summary, we demonstrated that MTMR14 deficiency led to adult obesity, metabolic dysfunction and inflammation with a normal chow diet, which was accompanied by the dysregulation of various inflammatory cytokines and adipokines and alterations in the PI3K/AKT and ERK signaling pathways in metabolic tissues. These results improve our understanding of the role of MTMR14 in fat accumulation, metabolism and inflammation and provide an important genetic target for the clinical diagnosis and treatment of adult obesity.

## Methods

### Mouse experiments

MTMR14^+/−^ mice (C57BL/6J background) were generously provided by Dr. Cheng-Kui Qu from Case Western Reserve University. All housing and experiments were performed in accordance with the Guide for the Care and Use of Laboratory Animals of South-Central University for Nationalities. We used heterozygous and heterozygous intercrosses to maintain the MTMR14^−/−^ mouse colony. The genotypes for each mouse were identified following the PCR genotyping method as described previously [14]. Experiments were conducted in a fasted state (except for the body weight experiment), with the food removed for 12 h (from 8 PM to 8 AM) and free access to water.

### Food intake and energy expenditure

Single WT or MTMR14 KO mice were housed in a cage containing a known amount of chow and new padding at 8 PM, and food was reweighed at 8 PM on the following day [44]. Daily food intake was calculated using the following formula:

$${\text{Daily food intake}} = {\text{food}}\,{\text{weight}}\,{\text{at}}\,8\,{\text{PM}} - {\text{food}}\,{\text{weight}}\,{\text{at}}\,8\,{\text{PM}}\,{\text{the}}\,{\text{following}}\,{\text{day}}$$

Energy expenditure was measured in age-matched fasted (12 h) mice as described previously [45]. Body weight changes were calculated using the following formula:

$${\text{Body weight change = }}\left(\frac{{{\text{fasted}}\,{\text{body}}\,{\text{weight}} - {\text{initial}}\,{\text{body}}\,{\text{weight}}}}{{{\text{initial}}\,{\text{body}}\,{\text{weight}}}} \right) \times 100\,\%$$

### Determination of TG and TC levels

TG and TC levels were determined in plasma using commercial kits from Biosystems (Barcelona, Spain). Blood samples for biochemical analysis were stored at −80 °C until use.

### Glucose tolerance tests (GTTs)

Age-matched WT and MTMR14 KO mice were fasted for 12 h with free access to water and subsequently injected intraperitoneally with 2 g d-glucose/kg (Sigma-Aldrich Co., St. Louis, MO, USA). Blood samples were taken from the tail vein prior to injection and at 15, 30, 60 and 120 min after injection. Glucose was measured using a glucometer (OneTouch UltraEasy, LifeScan Inc., Milpitas, CA, USA).

### Histological analyses

A portion of liver, muscle and fat from age-matched WT and MTMR14 KO mice was frozen, sectioned and stained using Oil Red O or hematoxylin and eosin (H&E). Pictures from each sample were obtained at 400× magnifications and analyzed.

### RNA extraction and real-time PCR

Total RNA was isolated from various organs using TRIzol reagent (Invitrogen, Carlsbad, CA, USA), and quantitative RT-PCR analysis was performed using the SYBR Green Master Mix (TOYOBO, Tsuruga, Japan) and the 7500 Fast QPCR system (Roche, Basel, Switzerland). The following primer sequences were used:

**Table:** 

Genes	Forward primers	Reverse primers
MTMR14	5’-AGACCTCATTCACCGAAGCA-3′	5′-TGTCACCACTCCGAAGAACA-3′
Actin	5′-AGAGGGAAATCGTGCGTGAC-3′	5′-CAATAGTGATGACCTGGCCGT-3′
Glut4	5′-CAGAGCTACAATGCAACG-3′	5′-GCCAATGAGAAAGGAAGAG-3′
Adiponectin	5′-AAGGACAAGGCCGTTCTCT-3′	5′-TATGGGTAGTTGCAGTCAGTTGC-3′
Leptin	5′-TCTCCGAGACCTCCTCCATCT-3′	5′-TTCCAGGACGCCATCCAG-3′
TNF-α	5′-TCTCAGCCTCTTCTCATTCCT-3′	5′-ACTTGGTGGTTTGCTACGAC-3′
IL-6	5′-CCTCTCTGCAAGAGACTTCCAT-3′	5′-AGTCTCCTCTCCGGACTTGT-3′
IL-1β	5′-CCCTGCAGCTGGAGAGTGTGGA-3′	5′-TGTGCTCTGCTTGTGAGGTGCTG-3′
MCP-1	5′-CTCAGCCAGATGCAGTTAACG-3′	5′-GGGTCAACTTCACATTCAAAGG-3′
PEPCK	5′-GGGCGGCTGGATGTCGGAAG-3′	5′-CCAATCTTGGCCAGCGGCGA-3′
G6P	5′-CTCTGGGTGGCAGTGGTCGGA-3′	5′-CAGGACCCACCAATACGGGCG-3′

### Serum analyses

Whole blood was collected from the orbit after a 12-h fast and centrifuged (1000×*g* for 15 min). Serum was isolated and analyzed using ELISAs for TNF-α (CUSABIO Biotech, Wuhan, China, CSB-E04741 m), IL-6 (CUSABIO, CSB-E04639m), leptin (CUSABIO, CSB-E04650 m) and adiponectin (CUSABIO, CSB-E07272m). Briefly, serum samples and standards were loaded in a 48-well microplate for incubation with captured antibodies, and detection antibodies were applied. Streptavidin-horseradish peroxidase (HRP) and tetramethyl benzidine chromogen were used to catalyze the color change reaction. Absorbance was measured at 450 nm.

### Western blotting

Tissues were harvested and lysed in RIPA buffer [50 mM Tris–HCl, (pH 7.5), 120 mM NaCl, 0.5 % Nonidet P-40 supplemented with a protease inhibitor cocktail (Roche) and PhosSTOP Phosphatase Inhibitor Cocktail (Roche)]. Lysates were clarified using centrifugation at 16,100×*g* for 15 min at 4 °C, and the supernatant was collected as the protein lysate. Proteins were resolved using SDS–polyacrylamide gel electrophoresis and transferred to Immobilon-P membranes (Millipore, Boston, MA, USA). Membranes were blocked in TBST (10 mM Tris [pH 8.0], 150 mM NaCl, 0.1 % Tween 20) supplemented with 5 % (wt/vol) powdered milk and incubated with primary antibodies (1:1000 dilution) at 4 °C overnight. The membranes were washed three times with TBST at room temperature and incubated with a horseradish peroxidase-conjugated secondary antibody (1:20,000 dilution) (GE Healthcare, Little Chalfont, Bucks, UK) for 1 h. Then, the membranes were washed with TBST three times, and signals were detected using ECL plus (GE Healthcare) according to the manufacturer’s protocol.

### Data analysis

Results are expressed as mean ± SD. Differences between groups were estimated for statistical significance using a multivariate analysis of variance (ANOVA) or 2-tailed Student’s t test, and *p* < 0.05 was considered statistically significant.

## Abbreviations

ANOVA: analysis of variance
ERKs: extracellular signal-regulated protein kinases
G6P: glucose-6-phosphate
Glut4: glucose transporter type 4
GTT: glucose tolerance test
IL-1β: interleukin 1β
IL-6: interleukin 6
KO: knock out
MCP-1: monocyte chemotactic protein 1
MTMR14: myotubularin-related protein 14
p-AKT: phosphorylation of AKT
p-ERK: phosphorylation level of ERK
PEPCK: phosphoenolpyruvate carboxykinase
PI3K/AKT: phosphoinositide 3 kinase/protein kinase B
PIPs: phosphatidylinositol phosphates
PTEN: phosphatase and tensin homolog
RYR: ryanodine receptor
WHO: World Health Organization
WT: wild type
SD: standard deviation
TC: total cholesterol
TG: triglyceride
TNF-α: tumor necrosis factor
